# Progressive Muscle Relaxation: intervention program in people with alcohol dependence

**DOI:** 10.1192/j.eurpsy.2023.1407

**Published:** 2023-07-19

**Authors:** T. Peralta, R. Zacarias, R. Lopes

**Affiliations:** 1 Serviço de Medicina Intensiva, Centro Hospitalar e Universitário de Coimbra; 2 Unidade de Alcoologia de Coimbra; 3 UCP Enfermagem de Saúde Mental e Psiquiátrica, Escola Superior de Enfermagem de Coimbra, Coimbra, Portugal

## Abstract

**Introduction:**

Alcohol consumption is often used in an attempt to reduce anxiety, being an inadequate coping strategy, it can lead to alcohol abuse and dependence.

Anxiety is a transient emotional state of reaction to situations perceived as threatening, frequent in people with alcohol dependence during the abstinence period.

Progressive Muscle Relaxation (PMR) is a technique that allows the person to reduce levels of stress, anxiety, anger and reach an increased state of calm.

**Objectives:**

To train people undergoing treatment for alcohol dependence to use the PMR after discharge.

To promote anxiety self-management.

To prevent relapse.

To evaluate the effect of the Therapeutic Relaxation Program (TRP) on the anxiety levels of people undergoing treatment for alcohol dependence.

**Methods:**

A TRP was conceived and implemented, based on Jacobson’s PMR, consisting of 6 sessions lasting 40 minutes, including 21 participants hospitalized for treatment of alcohol dependence.

Considering the inclusion criteria: clinical status favorable to participation; moderate or high level of anxiety; agree to participate voluntarily. Exclusion criteria: unfavorable clinical status (disorientation, confusion, agitation, delirium tremens, hypoacusis); level of mild anxiety or panic; not knowing how to read or write; refuse to participate voluntarily.

The experimental group (EG - in addition to the institution’s protocol treatment was included in the TRP) and the control group (CG - underwent the institution’s protocol treatment).

Participants gave informed consent.

In the TRP evaluation, the following were used: State-Trait Anxiety Inventory (STAI-form Y1) – before and after the intervention; physiological parameters (heart rate, respiratory rate and blood pressure) – before and after each session; and satisfaction questionnaire at the end of the program.

**Results:**

The evaluation of the physiological parameters showed a decrease after each relaxation session.

Comparing the mean values of the anxiety score (STAI-form Y1) between the two evaluation times (before and after the TRP), in the EG, there was a significant decrease in the anxiety scores, and this decrease was even greater in the group masculine.

In the CG, comparing the average values of the anxiety score (STAI-form Y1) in the same timings as in the EG, an increase in anxiety was verified.

The evaluation of the participants’ satisfaction revealed an increase in well-being and comfort at the end of the TRP, being greater in the male group.

**Conclusions:**

It is concluded that TRP produces positive effects in reducing anxiety levels, reducing physiological parameters and increasing the person’s well-being.

The TRP was effective in reducing the anxiety of people undergoing treatment for alcohol dependence, enabling them to use other coping tools/strategies that will contribute to maintaining alcohol abstinence and preventing relapse.

**Disclosure of Interest:**

None Declared

